# The Role of TNF-α in Neuropathic Pain: An Immunotherapeutic Perspective

**DOI:** 10.3390/life15050785

**Published:** 2025-05-14

**Authors:** Mario García-Domínguez

**Affiliations:** 1 Program of Immunology and Immunotherapy, CIMA-Universidad de Navarra, 31008 Pamplona, Spain; mgdom@unav.es; 2 Department of Immunology and Immunotherapy, Clínica Universidad de Navarra, 31008 Pamplona, Spain; 3 Centro de Investigación Biomédica en Red de Cáncer (CIBERONC), 28029 Madrid, Spain

**Keywords:** neuropathic pain, pro-inflammatory cytokine, TNF-α, nerve injury, immunotherapy

## Abstract

TNF-α is a pro-inflammatory cytokine that plays a pivotal role in the regulation of immune responses. It is predominantly produced by activated macrophages, although other cell types, such as T lymphocytes and NK cells, also contribute to its secretion. TNF-α participates in various physiological processes, including cell proliferation and differentiation. Moreover, TNF-α has been implicated in the pathogenesis of numerous inflammatory and autoimmune disorders. Recent studies have highlighted the important role of TNF-α in neuropathic pain, a complex and frequently disabling condition caused by nerve injury or dysfunction. Increased TNF-α levels in the nervous system have been associated with the onset of neuropathic pain, contributing to neuronal sensitization and alterations in pain signaling pathways. This study supports the idea that TNF-α connects the immune system with the nervous system, thereby supporting our understanding of the neuroimmune interface of pain and bringing a potential treatment against neuropathic pain: targeting TNF-α. Anti-TNF-α antibody administration reduces pain behaviors and neuroinflammation in preclinical animal models. Simultaneously, clinical trials are evaluating the safety and efficacy of anti-TNF-α treatments, with preliminary results indicating promising outcomes in patients experiencing neuropathic pain. Here, targeting TNF-α goes beyond its conventional spectrum of inflammatory pathologies and initiates a new mechanism-based approach to defining neuropathic pain, thereby improving the quality of life of the individuals affected and bringing together an area of colossal unmet clinical need.

## 1. Introduction

The International Association for the Study of Pain (IASP) defines pain as an un-pleasant sensory and emotional experience associated with or resembling that associated with actual or potential tissue damage [1,2]. Pain is a crucial symptom of diagnosis which usually signifies underlying illness to be addressed [3,4]. The stimulation of nociceptors, specialized neurons that play a role in the sensation of painful stimuli, leads to the feeling of pain [5]. Cell bodies of nociceptors are found in the sensory ganglia of the peripheral nervous system (PNS), e.g., trigeminal ganglia (TG) and dorsal root ganglia (DRG) [6]. To control pain perception and associated emotional responses, sensory neurons project to the spinal cord and brainstem where second-order neurons relay pain messages to the thalamus and other areas within the brain [7].

Pain may be divided based on various attributes, and one of these is its origin. Nociceptive pain is a result of tissue damage caused by physical trauma, surgery, or chemicals [8]. Nociplastic pain, whose name was derived in 2016, results from the abnormal processing of nociception without any detectable injuries in the tissues or visible lesions within the somatosensory system [9]. Finally, neuropathic pain results from damage or illness of the somatosensory system [10]. This pain is described as burning, tingling, or stinging and can be persistent even in the absence of visible tissue injury [11]. Neuropathic pain could be caused by a range of conditions such as metabolic diseases like diabetic peripheral neuropathy [12], viral illnesses such as post-herpetic neuralgia [13], autoimmune diseases like multiple sclerosis and Guillain–Barré syndrome [14,15], and chemotherapy-induced and inherited neuropathies [16,17].

Pain epidemiology yields insight into the global burden of the disease, differences in prevalence between population subgroups, as well as its consequences for public health [18,19]. Estimating the incidence of the condition worldwide is very difficult as there is no agreed international definition of neuropathic pain [20]. However, prevalence rates have been reported to be 8% in Europe [21], 15.7% in the United States of America [22], 17% in Canada [23], 3.2% in Japan [24], and 8.0% in China [25]. As one of the most common reasons for global disability, it underscores the demand for effective pain management and policies to strengthen health services [26].

The successful management of pain necessitates an extensive comprehension of its foundational causes. Medical professionals require specialized knowledge and tools to evaluate pain accurately by examining multiple dimensions including its intensity, temporal characteristics, and effects on everyday functions [27]. Effective pain management requires a patient-centered approach because it allows healthcare providers to tailor treatment strategies to each patient’s unique situation [28]. The implementation of a multidisciplinary approach demands the integration of both pharmacological treatments and psychological therapies [29].

The onset and progression of neuropathic pain has been linked to pro-inflammatory cytokines by numerous research studies [30]. Numerous immune cells such as lymphocytes, macrophages, dendritic cells, natural killer (NK) cells, mast cells, and stromal cells synthetize cytokines which are soluble proteins [31,32]. These proteins engage in the immune response while functioning as essential communicators within the immune system’s network [33]. An extensive array of scientific investigations has repeatedly shown a strong relationship between high pro-inflammatory cytokine levels and neuropathic pain patient discomfort severity [34,35]. The infection (or nerve injury)-prompted excessive release of pro-inflammatory cytokines such as TNF-α initiates pain [36].

The development and progression of numerous inflammatory and autoimmune diseases are significantly influenced by TNF-α, a major mediator of inflammation [37]. Activated macrophages, T cells, and NK cells produce human TNF-α, a homotrimeric protein with 157 αα (17 kDa) [38]. Other pro-inflammatory mediators, including interleukins [39], prostaglandins [40], leukotrienes [41], chemokines [42], and nitric oxide (NO) [43], are released as a result of TNF-α. There are three reasons why TNF-α is involved in pain modulation: (i) peripheral sensitization (TNF-α is involved in peripheral sensitization) [44], (ii) pain pathway activation (TNF-α strongly activates the ascending pain pathway via central sensitization) [45], and (iii) interaction with other pain mediators (TNF-α can potentiate the expression of other pain modulators) [46].

Moreover, because of its vital role in pain management, the immune system has attracted increased attention through its interactions with the nervous system [47]. Therefore, it is essential to enhance the immune system function to reduce the negative effects of pain. Monoclonal antibodies can be used in immunotherapy treatments to effectively treat a number of conditions associated with intense pain, including rheumatoid arthritis [48], migraine [49], osteoarthritis [50], chronic low back pain [51], and diabetic neuropathy [52].

This review aspires to report the recent and concise knowledge of TNF-α with particular reference to neuropathic pain pathophysiology and its potential therapeutic application. Through a review of preclinical and clinical research, this review seeks to further elucidate the mechanisms of action of TNF-α in pain sensitization, as well as determine the effectiveness of immunotherapeutic interventions in blocking TNF-α, specifically the monoclonal antibody approach. Concisely, this review will begin with a descriptive overview of the biological characteristics of TNF-α, followed by its involvement in the initiation and development of neuropathic pain, and conclude with a systematic consideration of anti-TNF-α antibodies as a therapeutic method for neuropathic pain.

## 2. Biology of TNF-α

Lloyd J. Old discovered TNF-α in the 1970s while studying these tumor regression-related factors [53]. This pro-inflammatory cytokine, which is produced by macrophages, is crucial for the control of inflammation, apoptosis, and the immune system [37]. TNF-α plays a key role in the pathophysiology of several autoimmune diseases, including rheumatoid arthritis [49] and chronic low back pain [51].

### 2.1. Characteristics of TNF-α

**Figure 1:**
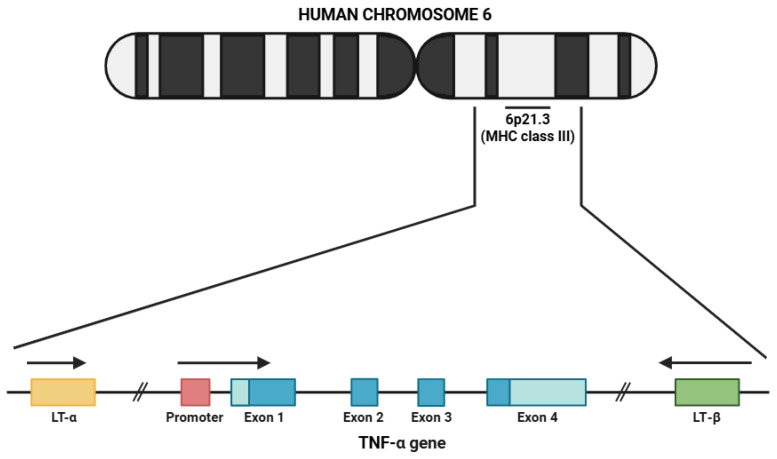
Schematic representation of the genomic localization of the human TNF-α gene. Abbreviations: MHC (major histocompatibility complex), LT-α (lymphotoxin alpha), TNF-α (tumor necrosis factor alpha), and LT-β (lymphotoxin beta).

TNF-α is a cytokine that belongs to the TNF superfamily. Its gene (Figure 1) is located on the short arm of human chromosome 6 at position 6p21.3, specifically in the major histocompatibility complex (MHC) class III region, and is larger than 3 kb. There are four exons in the TNF-α gene, with the structural conformation allowing for the subsequent transcription and translation of TNF-α into its final form [54]. In rats, the TNF-α gene has been mapped onto chromosome 20q12 with a comparable exon–intron structure compared to that found in humans [55]. The TNF-α gene has been mapped to chromosome 17 in mice again within the region of the MHC, thus highlighting the evolutionary conservation of TNF-α across numerous mammalian species [56]. The structural and functional homology of the TNF-α protein between these species renders translational research employing rodent models to study its role in inflammatory and immune-mediated diseases possible [57].

**Figure 2:**
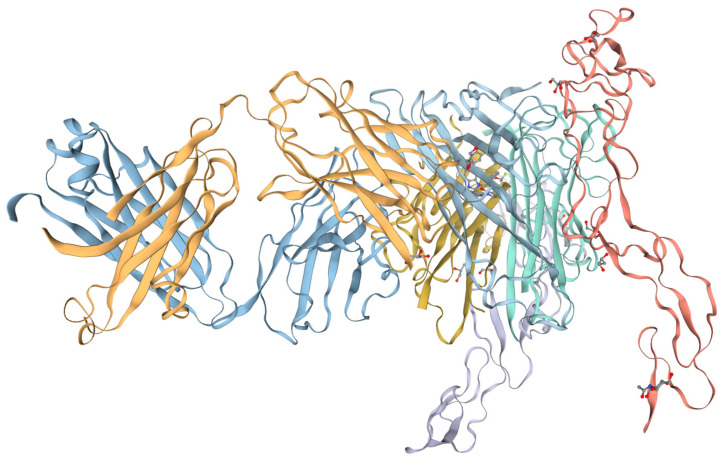
Tridimensional structure of human TNF-α. Image generated using the ExPASy software.

The primary translation product of the TNF-α gene is a precursor protein called pro-TNF-α. The precursor protein is a 26 kDa type II transmembrane protein consisting of 233 αα [58]. While it is being synthesized, pro-TNF-α is inserted into the plasma membrane where it is oligomerized into a homotrimeric complex [59]. Upon being incorporated into the cell membrane, pro-TNF-α is cleaved by the TNF-α converting enzyme (TACE), a metalloprotease of the ADAM family [60]. The result of this cleavage is the shedding of the mature soluble form of TNF-α with a molecular weight of 17 kDa and 157 αα [38]. The mature TNF-α maintains the trimeric structure in the extracellular environment, enabling it to bind to cognate receptors TNFR1 and TNFR2 and induce a chain reaction of intracellular signaling processes [61]. In summary, from a structural standpoint, the TNF-α molecule (Figure 2) adopts a distinctive homotrimeric architecture that underlies its biological activity.

TNF-α consists of a trimeric assembly of three identical monomeric subunits [62]. The three-dimensional structure of a single TNF-α monomer is imitated from a β-sandwich fold, which consists of two antiparallel β-sheets closely superimposed over each other in a particular pattern, which serves to enhance the overall stability as well as the functional efficiency of the molecule [63]. TNF-α is a homotrimer composed of three identical monomeric subunits, a feature associated with antiparallel β-sheets [63]. The β-sandwich fold is very compact, and this keeps all of the monomers in a rigid and stable form, a feature necessary for proper receptor binding [64].

The β-sandwich fold offers the structural framework upon which TNF-α may adopt a particular orientation in its trimeric form. In the homotrimer form, the monomers are symmetrically arranged, aligning their β-sheets so that the central region of the β-sandwiches forms the core of the structure [65]. The hydrophobic core of each monomer is stabilized by a network of nonpolar contacts, whereas the outer surface of the trimer is composed of both hydrophobic and hydrophilic residues that are involved in receptor binding [66,67].

### 2.2. TNF-α Receptor: Structure and Localization

TNF-α exerts its effects by binding to two distinct receptors: the 55 kDa type I tumor necrosis factor receptor (TNFR1), which is present in all human tissues [68], and the 75 kDa type II tumor necrosis factor receptor (TNFR2), which is present in immune cells, neurons, and endothelial cells [69,70,71]. These receptors possess intracellular domains that are structurally and functionally distinct, enabling the recruitment of distinct adaptor proteins that trigger different signaling cascades.

**Figure 3:**
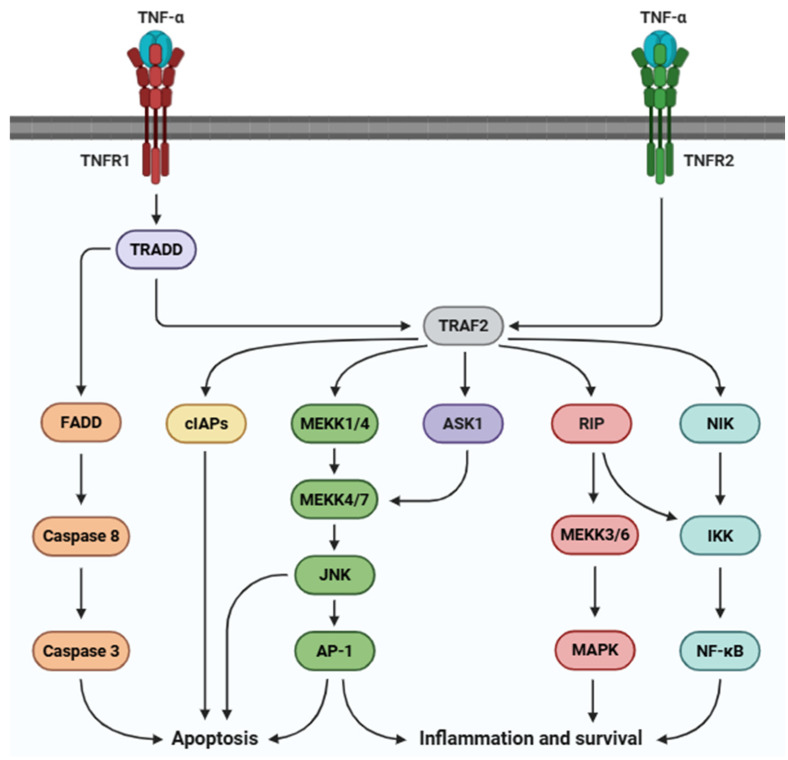
Signaling pathways mediated by TNFR1 and TNFR2. Abbreviations: TNF-α (tumor necrosis factor alpha), TNFR1 (tumor necrosis factor receptor 1), TNFR2 (tumor necrosis factor receptor 2), TRADD (TNF receptor-associated death domain), TRAF2 (TNF receptor-associated factor 2), FADD (Fas-associated death domain), cIAPs (cellular inhibitor of apoptosis proteins), MEKK1/4 (mitogen-activated protein kinase/ERK kinases 1 and 4), MEKK4/7 (mitogen-activated protein kinase/ERK kinases 4 and 7), JNK (c-Jun N-terminal kinase), AP-1 (activator protein 1), ASK1 (apoptosis signal-regulating kinase 1), RIP (receptor-interacting protein), MEKK3/6 (mitogen-activated protein kinase/ERK kinases 3 and 6), MAPK (mitogen-activated protein kinase), NIK (NF-κB-inducing kinase), IKK (IκB kinase), and NF-κB (nuclear factor kappa-light-chain-enhancer of activated B cells).

TNFR1 (Figure 3) is a central regulator of several intracellular pathways of immune responses, including neutrophil migration, complement system activation, and cytokine release [72,73]. Upon TNF-α binding, TNFR1 recruits TRADD, RIPK1, and FADD, leading to the activation of JAK (JAK1 and JAK2) and STAT proteins (STAT3 and STAT5) [74,75,76,77,78]. Additionally, the MAPK and NF-κB pathways trigger a cascade of molecular events that lead to the transcription of a wide array of genes involved in the regulation of inflammation, immune responses, and cell survival. The MAPK pathway, through its subfamilies (e.g., ERK, JNK, and p38 MAPK), plays a key role in the activation of transcription factors that control cell responses to stress, growth stimuli, and inflammatory stimuli [79,80,81]. Specifically, these pathways lead to the activation of transcription factors such as AP-1 and Elk-1 that enhance the production of pro-inflammatory cytokines, chemokines, and enzymes involved in the recruitment of immune cells and tissue remodeling [82,83]. On the other hand, NF-κB activation, primarily through the p65/p50 dimer, is a central mediator of the inflammatory response, inducing the transcription of those genes encoding cytokines IL-1β and IL-6, as well as other pro-apoptosic molecules such as caspase 3 [84,85,86].

In contrast, TNFR2-mediated signaling (Figure 3) is facilitated by several interactions with TRAF1 and TRAF2 [87,88]. Unlike TNFR1, TNFR2 lacks a death domain and is primarily linked to cell survival and immune regulation rather than apoptosis [89]. TRAF2, notably, activates JNK through ASK1, leading to the activation of NF-κB and the subsequent transcription of some pro-inflammatory genes, such as IL-1β [90], IL-6 [90], CXCL1 [91], and COX-2 [92].

### 2.3. Biological Roles of TNF-α

One of the primary functions of TNF-α is its role in orchestrating the innate immune response. As a central mediator, TNF-α facilitates the recruitment and activation of neutrophils, dendritic cells, and T lymphocytes, all of which are critical for combating bacterial, viral, and fungal pathogens [93,94]. Through the activation of several signaling pathways, TNF-α induces the expression of some chemokines (like CXCL10 and CCL2), adhesion molecules (such as ICAM-1 and VCAM-1), and pro-inflammatory mediators (such as prostaglandins and NO), thus enhancing vascular permeability and promoting immune cell extravasation to sites of infection or tissue injury [95,96,97]. Beyond its role in pathogen clearance, TNF-α also contributes to immune homeostasis by regulating macrophage polarization and antigen presentation [98].

Beyond its role in acute inflammation, TNF-α is crucial in the regulation of adaptive immunity, particularly in the differentiation and function of Th cells [99]. It exerts a pivotal influence on the polarization of the Th1 and Th17 subsets, which are critical for mounting effective immune responses against intracellular pathogens [100]. TNF-α also plays a key role in autoimmune diseases, such as rheumatoid arthritis, inflammatory bowel disease, and psoriasis, where its aberrant activity contributes to chronic inflammation and tissue damage [101,102,103]. Moreover, TNF-α enhances B cell activation and antibody production, further reinforcing its role in adaptive immune responses [104].

Alternatively, TNF-α exhibits a dual role in cancer, acting as both a tumor suppressor and a tumor promoter depending on the context. Under particular conditions, TNF-α promotes apoptosis in cancer cells via the activation of caspase-dependent pathways [105]. However, chronic TNF-α signaling can foster tumor progression by promoting angiogenesis, epithelial–mesenchymal transition, and immune evasion mechanisms [106,107,108]. Additionally, TNF-α enhances the tumor microenvironment by recruiting immunosuppressive cells such as myeloid-derived suppressor cells (MDSCs) and Tregs, thereby facilitating tumor immune escape [109,110].

In the context of metabolic diseases, TNF-α has been implicated in insulin resistance and obesity-related inflammation. This cytokine interferes with insulin receptor signaling by activating serine kinases that phosphorylate insulin receptor substrate (IRS) proteins, leading to diminished insulin sensitivity and contributing to the development of type 2 diabetes mellitus [111,112]. Increased levels of TNF-α in adipose tissue further exacerbate metabolic dysfunction by promoting lipolysis, hepatic gluconeogenesis, and systemic inflammation [113].

On the other hand, TNF-α is a critical player in neuroinflammatory processes associated with neurodegenerative diseases including Alzheimer’s disease, Parkinson’s disease, and multiple sclerosis [114,115,116]. Dysregulated TNF-α expression drives chronic neuroinflammation, glial activation, and neuronal apoptosis, aggravating the progression of the disease [117]. In Alzheimer’s disease, TNF-α has been linked to amyloid-beta (Aβ) plaque accumulation and synaptic dysfunction [118], whereas in Parkinson’s disease, TNF-α-mediated microglial activation leads to dopaminergic neuron degeneration [119].

Despite its pathogenic roles, TNF-α is critical for tissue repair and regeneration. This cytokine aids angiogenesis, fibroblast proliferation, and extracellular matrix remodeling, facilitating wound healing [120]. However, excessive TNF-α activity can result in chronic wounds and fibrosis, as seen in conditions such as diabetic ulcers and pulmonary fibrosis [121,122].

## 3. Role of TNF-α in Neuropathic Pain

**Figure 4:**
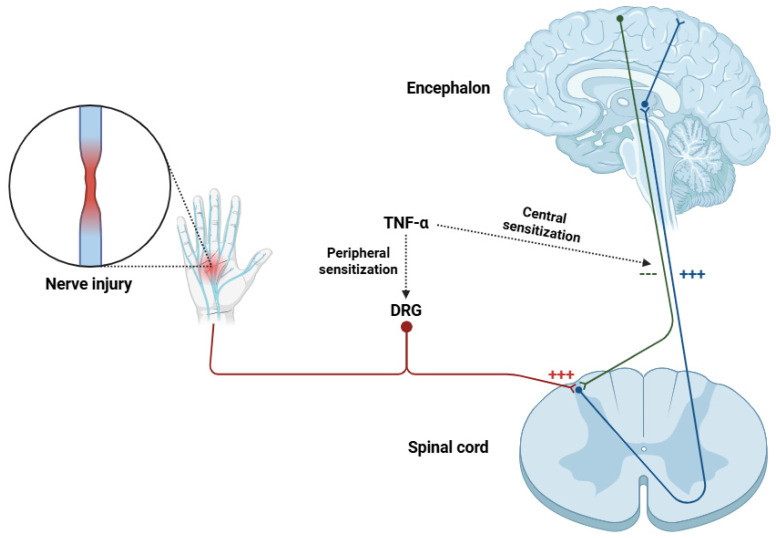
Diagram showing the cascade of events after TNF-α activation due to peripheral nerve injury. TNF-α causes the release of large amounts of CGRP from the dorsal horn so that transmission in the ascending pain pathway is facilitated. This process massively inhibits the neuronal activity of descending pain control, thereby facilitating pain perception. Abbreviations: TNF-α (tumor necrosis factor alpha) and DRG (dorsal root ganglia).

TNF-α, a pro-inflammatory cytokine, enhances nociceptive signaling and sensitizes primary afferent neurons, contributing to neuropathic pain (Figure 4) [123]. In some neuropathic pain models, TNF-α (and its receptors TNFR1 and TNFR2) show increased expression and activity in the PNS. Peripheral actions of TNF-α involve highly complex cellular and molecular interactions, leading to hyperexcitability in nociceptors [124].

One of the main mechanisms by which TNF-α produces peripheral sensitization is through the modulation of voltage-gated sodium channels (VGSCs) in the dorsal root ganglia (DRG) [125]. TNF-α leads to upregulation of Na_V_1.3, Na_V_1.7, and Na_V_1.8, which play central roles in nociceptive transmission [126,127]. Overexpression leads to increased neuronal excitability, facilitating the generation and propagation of many action potentials in primary afferent neurons [128]. The outcome of this process is a strong intensification of pain signaling, causing hyperalgesia and mechanical allodynia [129]. Perisciatic injection of exogenous TNF-α has been reported to induce chronic mechanical allodynia, a demonstration of its role as a central mediator of chronic pain in the periphery [130]. Beyond its influence on VGSCs, TNF-α directly regulates other ion channels, a crucial process in nociceptive signaling [131]. TNF-α enhances the function of transient receptor potential ankyrin 1 (TRPA1) [132] and transient receptor potential vanilloid 1 (TRPV1) [133], which are essential mediators of pain perception in primary sensory neurons. TNF-α also modulates voltage-gated calcium channels (VGCCs), specifically the Ca_V_3.2 subunit, which is involved in low-threshold calcium currents in sensory neurons [134]. The augmentation of Ca_V_3.2 activity by TNF-α enhances calcium intake, which in turn causes increased release of neurotransmitters from nociceptive terminals [135].

In the spinal cord (Figure 4), TNF-α plays key roles in excitatory synaptic transmission by regulating the expression, trafficking, and phosphorylation of the principal ionotropic glutamate receptors [136,137]. Notably, TNF-α enhances synaptic transmission via AMPA and NMDA receptors to improve excitatory postsynaptic currents (EPSCs) [138]. This potentiation of excitatory synapses is a determinant of pain signaling amplification and central sensitization generation for chronic pain disorders [139].

The synaptic effects of TNF-α are primarily exerted by stimulating intracellular signal transduction cascades such as the NF-κB, p38 MAPK, and JNK pathways [140,141]. The stimulation of these signaling pathways results in the phosphorylation of NMDA receptor subunits, notably NR1 and NR2, thereby increasing their conductance and synaptic insertion [142]. Post-translational modification of NMDA receptors via phosphorylation is responsible for the synaptic plasticity mechanisms underlying hyperalgesia and allodynia that characterize neuropathic pain states [143]. Furthermore, TNF-α promotes synaptic insertion of calcium-permeable AMPA receptors via the modulation of the expression of GluA1 subunits, further enhancing the excitability of the neuron [144]. The protein kinase C (PKC) and protein kinase A (PKA) signaling pathways also control the TNF-α-mediated AMPA receptor trafficking and promote the phosphorylation of GluA1 at serine 831 and serine 845, respectively. This phosphorylation allows for the insertion of AMPA receptors into the synapse, increasing excitatory synaptic transmission [144,145,146]. TNF-α modulates synaptic activity by acting on astrocytes, increasing glutamate release through the activation of connexin 43 hemichannels and playing a role in excitotoxicity and heightened sensitivity to pain intensity [147].

Apart from its direct effect on excitatory neurotransmission, TNF-α also disrupts the delicate balance between excitatory and inhibitory signaling processes in the spinal cord. TNF-α suppresses inhibitory synaptic transmission by downregulating GABA and glycine-mediated neurotransmission [148,149]. This suppression is evoked by the activation of TNFR1, which promotes downstream MAPK signaling in interneurons [148,149]. The resulting reduction in inhibitory tone results in disinhibition, thereby increasing central sensitization and pain perception [150].

TNF-α is also a key mediator in neuroinflammation by stimulating spinal cord microglia [151]. Activated microglia also promote the secretion of other pro-inflammatory mediators, including interleukin-1β (IL-1β) and TNF-α, to generate a self-reinforcing feedforward loop of neuroimmune responses [152]. This neuroinflammatory process enhances neuronal excitability, augments maladaptive synaptic plasticity, and causes chronicity of pain [153]. Furthermore, microglial activation facilitates the production of reactive oxygen species (ROS) and nitric oxide (NO), which improve neuronal dysfunction as well as oxidative stress in spinal cord microenvironments [154,155].

Beyond the spinal cord, TNF-α upregulation is seen in a number of supraspinal regions involved in pain modulation, including the ventrolateral periaqueductal gray, hippocampus, and locus coeruleus (Figure 4) [156,157,158]. All of these brain regions are not only involved in descending pain inhibition and facilitation but also in cognitive and affective processing [159,160,161]. The discovery of increased levels of TNF-α in these regions indicates that this cytokine links nociceptive input and affective responses, thereby contributing to the complexity of neuropathic pain [124]. In the ventrolateral periaqueductal gray, TNF-α imbalance disrupts the harmony between glutamatergic and GABAergic transmission, thereby inhibiting the endogenous descending pain modulation system [156]. In the locus coeruleus, TNF-α modulates noradrenergic transmission by altering the function of noradrenaline transporters, with effects on pain modulation and stress behavior [162]. In the hippocampus, TNF-α neuroinflammation has been linked to synaptic remodeling alterations and neuroplasticity disruption [163]. Via interaction with glial cells, TNF-α generates a neurotoxic environment that fosters synaptic dysfunction and cognitive decline [164]. This neuroinflammatory response may explain the cognitive deficits commonly observed in patients with neuropathic pain, including impairments in memory and attentional processing [165].

Also, TNF-α-mediated neuroinflammation in such supraspinal structures can play a critical role in comorbid depression–anxiety development in neuropathic pain [166]. The modulation of serotonergic and dopaminergic neurotransmission has been implicated in the regulation of mood with TNF-α [167,168]. TNF-α induction of the kynurenine pathway, which diverts tryptophan metabolism from serotonin synthesis to the neurotoxic metabolite quinolinic acid, can contribute to the pathophysiology of pain affective disorders [169].

All of these observations underscored the key role of TNF-α to regulate neuropathic pain via diverse molecular processes, like ion channel functions, synaptic plasticity, neuroinflammation, and central disinhibition. The clarification of forthcoming investigations intended to delineate TNF-α signal transduction cascades and downstream effectors may unravel new pharmacologic approaches to counter neuropathic pain and its related neuropsychiatric repercussions.

## 4. Therapeutic Targeting of TNF-α in Neuropathic Pain

Immunotherapy is a new treatment strategy that is used to enhance or regulate immune system function to treat a wide range of diseases, such as cancer [170], autoimmune conditions [171], and neuropathies [172]. Immunotherapy is the form of therapy that takes advantage of innate immunity mechanisms to restore immune surveillance and normalize abnormal immune responses, offering more targeted and less harmful substitutes for conventional therapies like chemotherapy and/or corticosteroids [173,174]. Not only is immunotherapy linked with an improved safety profile, but it has also shown clear evidence of benefit in preclinical models and in clinical studies [175].

Abundant immune mediators discussed in relation to neuropathies point to TNF-α as an important pro-inflammatory cytokine. Experimental and clinical research is utilized to determine its important role towards the initiation and establishment of neuropathic states in which it is involved in neuroinflammation, neuronal injury, and pain sensitivity. Therefore, blocking TNF-α (and other pro-inflammatory cytokines) is a potential therapeutic approach for blocking, if not reversing, the establishment of neuropathic pain.

A comprehensive overview of the most relevant immunotherapies currently investigated by preclinical research is found in Table 1. This information has invariably reflected the promising therapeutic potential of TNF-α-based immunotherapy for the management of neuropathic syndromes.

Immunotherapeutics are discussed in Table 2, which identifies monoclonal antibodies that have advanced to clinical trials or gained clinical approval. All of these antibodies directly block TNF-α through a unique mechanism, thereby providing evidence for immunotherapy against neuropathic pain. A systematic review was performed to find randomized controlled clinical trials using anti-TNF-α in patients with neuropathic pain in the principal medical databases such as PubMed between 2003 and 2023. Inclusion criteria involved randomized controlled trials on the administration of anti-TNF-α in patients suffering from neuropathic pain. This period was selected to investigate the use of monoclonal antibodies targeting TNF-α due to significant scientific advancements, the increasing number of clinical studies, and notable progress in regulatory developments.

## 5. Conclusions

Neuropathic pain is a complex disease resulting from a lesion or a dysfunction in the somatosensory nervous system. Its pathophysiology is the result of various molecular and cellular mechanisms, one of which is of particular interest: inflammation. Indeed, TNF-α, a pro-inflammatory cytokine, has been of particular interest in recent years because of its role in modulating nociceptive transmission and in causing peripheral and central sensitization. TNF-α is one of the key neuroinflammatory mediators, whose action on its receptors, TNFR1 and TNFR2, results in its downstream activities. Upon activation, TNF-α generates cascades of signals that alter the excitability of neurons, stabilize synaptic transmission, and stimulate glial cell activation. All of these evoke maladaptive plasticity, sensitizing pain and maintaining chronic pain. Inside the PNS, TNF-α sensitizes nociceptors by activating ion channel function and increasing neuronal excitability. Inside the CNS, TNF-α increases microglial and astrocytic activation that generates a pro-inflammatory environment which sustains increased pain levels.

Since it acquires a critical function in neuropathic pain pathogenesis, TNF-α is also considered a desirable therapeutic target. In preclinical studies, evidence showed that reducing behavior of pain occurred when pharmacologic blockage of TNF-α was conducted through monoclonal antibodies in various animal models. Clinical trials explored the therapeutic value of monoclonal anti-TNF-α treatments, with some documenting significant relief of pain in rheumatoid arthritis, ankylosing spondylitis, and chronic radiculopathy. Such positive results are not transferable to the population of neuropathic pain due to varying responses from patients, side effects, and most importantly, differing methods with which to assail.

However, some limitations need to be specified in relation to this review. First, while this article does not constitute a formal meta-analysis, it offers a comprehensive overview of selected studies. Second, the majority of evidence implicating TNF-α in neuropathic pain, as well as the rationale for anti-TNF-α therapies, is primarily derived from preclinical models, which are not probably optimal for representing human neuropathic pain conditions; additionally, the pharmacodynamics and pharmacokinetics of monoclonal anti-TNF-α antibodies are unclear, so available data remain limited about the optimal dosing or long-term safety profile of these antibodies. Moreover, trial design and patient population heterogeneity appear to be the main pathway to obtain final conclusions about the utility of anti-TNF-α therapy for the treatment of neuropathic pain. These aspects underscore the need for targeted and more rigorous research aligned with this objective.

Significant gaps remain in knowledge about the multifunctional role that TNF-α plays in neuropathic pain, despite the highly promising evidence. Future research should focus on elucidating the role of TNF-α signaling (by TNFR1 and TNFR2) that induces physiologically different and typically antagonistic effects. Further effort is required to build a valuable battery of biomarkers of response to therapy in order to support the implementation of customized therapeutic approaches. Additional efforts are needed to clarify the long-term safety and efficacy of anti-TNF-α therapy, especially its potential influences on neuroinflammatory homeostasis.

In summary, increased understanding of the role of this cytokine in neuropathic pain should result in improved treatment techniques and the development of effective strategies for pain management. Through the unification of molecular neuroscience, immunology, and clinical pharmacology within a single body of activity, future scientists may be able to determine more effective targeted interventions for pain relief with fewer side effects to the benefit of improved patient outcomes and quality of life.

## Figures and Tables

**Table 1:** List of anti-TNF-α treatments employed in preclinical models. Abbreviations: DPN (diabetic polyneuropathy), TNF-α (tumor necrosis factor alpha), MNCV (motor nerve conduction velocity), SNCV (sensory nerve conduction velocity), IENFD (intraepidermal nerve fiber density), NF-κB p65 (nuclear factor kappa-light-chain-enhancer of activated B cells, p65 subunit), DRG (dorsal root ganglia), CCI (chronic constriction injury), PST (partial sciatic nerve transection), SCI (sciatic nerve injury), CIPN (chemotherapy-induced neuropathy), and BTZ (bortezomib).

Type of Neuropathy	Species Tested	Effects	References
Diabetic polyneuropathy (DPN)	Mice	TNF-α^+/+^ diabetic mice exhibited significant impairments in motor and sensory nerve conduction velocities (MNCV and SNCV), tail flick responses, and intraepidermal fiber density (IENFD), along with elevated expression of NF-κB p65 and cleaved caspase-3 in their DRGs	[176]
Chronic constriction injury (CCI)	Mice	Suppressed thermal hyperalgesia and mechanical allodynia	[177]
	Mice	Suppressed thermal hyperalgesia and mechanical allodynia	[178]
	Mice	Suppressed thermal hyperalgesia and mechanical allodynia	[179]
	Rats	Perioperative anti-TNF-α treatment modulated the inflammation and fibrosis associated with CCI. Morphometric and immunohistochemical analyses demonstrated that a single systemic administration of anti-TNF-α attenuated early inflammatory responses	[180]
	Rats	Suppressed thermal hyperalgesia and mechanical allodynia	[181]
Partial sciatic nerve transection (PST)	Mice	Suppressed thermal hyperalgesia and mechanical allodynia	[178]
Sciatic nerve injury (SCI)	Mice	Suppressed mechanical allodynia	[182]
	Mice	Suppressed mechanical allodynia	[183]
Chemotherapy-induced neuropathy (CIPN)	Mice	Treatment with anti-TNF-α significantly preserved the sensory nerve action potential amplitude and prevented the loss of myelinated and unmyelinated fibers in bortezomib (BTZ)-induced neurotoxicity	[184]
Dengue virus-induced neuropathy	Mice	Mitigated encephalitis	[185]

**Table 2:** Overview of anti-TNF-α therapies utilized in clinical practice. Abbreviations: TNF-α (tumor necrosis factor alpha), SUCRA (surface under the cumulative ranking curve), CRPS (complex regional pain syndrome), IL-6 (interleukin 6), ISS (injury severity score), EuroQol (European quality of life), EQ VAS (EuroQol visual analog scale), ITT (intent-to-treat), PP (per protocol), IQR 1–2 (interquartile range of 1 to 2), and SSFN (sarcoidosis-associated small fiber neuropathy).

Type of Neuropathy	Drug Employed	Effects	References
Postherpetic neuralgia	Infliximab Adalimumab	A retrospective review across 12 dermatology clinics evaluated herpes zoster patients treated with TNF-α inhibitors, including infliximab and adalimumab. The analysis revealed a reduced incidence of postherpetic neuralgia in this cohort.	[186]
Sciatica	Infliximab	Within one hour of infusion, leg pain was reduced by 50%. After two weeks, 60% of patients receiving infliximab were pain-free, compared to 16% in the control group. This therapeutic effect persisted at three months, with 90% of infliximab-treated patients remaining pain-free vs. 46% in the control group.	[187]
	Infliximab	A 3 mg/kg dose of infliximab provided sustained improvement in leg pain and disability over one year. Neurologic abnormalities improved in the infliximab group, although disk herniation volume reduction was very similar between groups.	[188]
	Adalimumab	Leg pain improved more significantly over time in the adalimumab group compared to the placebo group, although the effect size was modest. A significantly higher proportion of patients in the adalimumab group met the criteria for “responders” and “low residual disease impact”. Additionally, fewer surgical discectomies were required in the adalimumab group.	[189]
	Infliximab Adalimumab	Intravenous anti-TNF-α ranked highest for leg pain relief and subcutaneous anti-TNF-α ranked highest for lumbar pain relief, based on SUCRA analysis. All treatments had medium to high safety rankings in terms of withdrawal rates.	[190]
Complex Regional Pain Syndrome (CRPS)	Infliximab	A significant decrease in local concentrations of TNF-α and IL-6 in blister fluid was observed. There was a slight improvement in clinical signs, with reductions in pain, temperature, and edema, as well as improved motor function. The patients also reported an overall improvement in well-being. By the end of the treatment clinical symptoms showed improvement.	[191]
	Infliximab	There was no significant difference in the total ISS score between the treated and control groups. However, a trend was observed, suggesting a greater reduction in TNF-α in the infliximab group compared to the placebo group. Additionally, a subscale of the EuroQol (EQ VAS) showed a significant decrease in health status in the intervention group compared to the placebo group.	[192]
	Adalimumab	Three patient subgroups were identified, each consisting of three patients: “nonresponders”, “partial responders”, and “robust responders”, with the latter group showing improvement in nearly all parameters. Both the ITT and PP analyses revealed only a trend toward improvement in mechanical pain thresholds following treatment.	[193]
	Adalimumab	The infliximab dosage is 5 mg/kg, administered every four to six weeks. A total of 7 patients (of 15) completed a global perceived effect survey, all reporting improvement (IQR 1–2).	[194]
Sarcoidosis-associated small fiber neuropathy (SSFN)	Infliximab	SSFN was diagnosed in 143 individuals, with 28 cases having other neuropathy causes. Pain and paresthesias were the most common symptoms, with 54% being non-length-dependent. Dysautonomia was present in 61 patients, mainly with cardiac symptoms. Symptomatic improvement was seen in 8 of 12 with infliximab. A total of 4 of 27 untreated patients showed improvement.	[195]

## Data Availability

Not applicable. No new data were generated.

## Abbreviations

The following abbreviations are used in this manuscript:

ADAM	A disintegrin and metalloprotease
AMPA	α-amino-3-hydroxy-5-methyl-4-isoxazolepropionic acid
AP-1	Activator protein 1
ASK1	Apoptosis signal-regulating kinase 1
BTZ	Bortezomib
Ca_V_3.2	Voltage-gated calcium channel 3.2
CCI	Chronic constriction injury
CCL2	C-C Motif chemokine ligand 2
CGRP	Calcitonin gene-related peptide
cIAP	Cellular inhibitor of apoptosis protein
CIPN	Chemotherapy-induced peripheral neuropathy
CNS	Central nervous system
COX-2	Cyclooxygenase 2
CRPS	Complex regional pain syndrome
CXCL1	C-X-C Motif Chemokine Ligand 1
CXCL10	C-X-C Motif Chemokine Ligand 10
DPN	Diabetic polyneuropathy
DRG	Dorsal root ganglia
Elk-1	ETS like-1 protein
EPSC	Excitatory postsynaptic current
EQ VAS	EuroQol visual analog scale
ERK	Extracellular signal-regulated kinase
EuroQol	European quality of life
FADD	Fas-associated death domain
GABA	Gamma-aminobutyric acid
GluA1	Glutamate receptor subunit 1 (AMPA receptor)
IASP	International Association for the Study of Pain
ICAM-1	Intercellular adhesion molecule 1
IENFD	Intraepidermal nerve fiber density
IKK	IκB kinase
IL-1β	Interleukin 1 beta
IL-6	Interleukin 6
IQR 1–2	Interquartile range of 1 to 2
IRS	Insulin receptor substrate
ISS	Injury severity score
ITT	Intent-to-treat
JAK1	Janus kinase 1
JAK2	Janus kinase 2
JNK	c-Jun N-terminal kinase
LT-α	Lymphotoxin alpha
LT-β	Lymphotoxin beta
MAPK	Mitogen-activated protein kinase
MDSC	Myeloid-derived suppressor cell
MEKK1/4	Mitogen-activated protein kinase/ERK kinases 1 and 4
MEKK3/6	Mitogen-activated protein kinase/ERK kinases 3 and 6
MEKK4/7	Mitogen-activated protein kinase/ERK kinases 4 and 7
MHC	Major histocompatibility complex
MNCV	Motor nerve conduction velocity
Na_V_1.3	Voltage-gated sodium channel 1.3
Na_V_1.7	Voltage-gated sodium channel 1.7
Na_V_1.8	Voltage-gated sodium channel 1.8
NF-κB	Nuclear factor kappa-light-chain-enhancer of activated B cells
NF-κB p65	Nuclear Factor kappa-light-chain-enhancer of activated B cells, p65 subunit
NIK	NF-κB-inducing kinase
NK	Natural killer cell
NMDA	N-methyl-D-aspartate
NO	Nitric oxide
NR1	NMDA receptor subunit 1
NR2	NMDA receptor subunit 2
p38 MAPK	p38 mitogen-activated protein kinase
p50	NF-κB p50 subunit
p65	NF-κB p65 (RelA) subunit
PKA	Protein kinase A
PKC	Protein kinase C
PNS	Peripheral nervous system
PP	Per protocol
pro-TNF-α	Pro-tumor necrosis factor alpha
PST	Partial sciatic nerve transection
RIP	Receptor-interacting protein
RIPK1	Receptor-interacting serine/threonine-protein kinase 1
ROS	Reactive oxygen species
SCI	Sciatic nerve injury
SNCV	Sensory nerve conduction velocity
SSFN	Sarcoidosis-associated small fiber neuropathy
STAT	Signal transducer and activator of transcription
STAT3	Signal transducer and activator of transcription 3
STAT5	Signal transducer and activator of transcription 5
SUCRA	Surface under the cumulative ranking curve
TACE	TNF-α converting enzyme
TG	Trigeminal ganglia
Th	T helper cell
Th1	T helper cell type 1
Th17	T helper cell type 17
TNF	Tumor necrosis factor
TNFR1	Tumor necrosis factor receptor 1
TNFR2	Tumor necrosis factor receptor 2
TNF-α	Tumor necrosis factor alpha
TRADD	TNF receptor-associated death domain
TRAF1	TNF receptor-associated factor 1
TRAF2	TNF receptor-associated factor 2
Treg	Regulatory T cell
VCAM-1	Vascular cell adhesion molecule 1
VGCC	Voltage-gated calcium channel
VGSC	Voltage-gated sodium channel
